# Progressive Abduction Loading Therapy with Horizontal-Plane Viscous Resistance Targeting Weakness and Flexion Synergy to Treat Upper Limb Function in Chronic Hemiparetic Stroke: A Randomized Clinical Trial

**DOI:** 10.3389/fneur.2018.00071

**Published:** 2018-02-19

**Authors:** Michael D. Ellis, Carolina Carmona, Justin Drogos, Julius P. A. Dewald

**Affiliations:** ^1^Department of Physical Therapy and Human Movement Sciences, Feinberg School of Medicine, Northwestern University, Chicago, IL, United States; ^2^Department of Biomedical Engineering, McCormick School of Engineering, Northwestern University, Chicago, IL, United States; ^3^Department of Physical Medicine and Rehabilitation, Feinberg School of Medicine, Northwestern University, Chicago, IL, United States

**Keywords:** stroke, stroke rehabilitation, upper extremity, robotics, physical and rehabilitation medicine, physical therapy modalities, exercise therapy, resistance training

## Abstract

**Background:**

Progressive abduction loading therapy has emerged as a promising exercise therapy in stroke rehabilitation to systematically target the loss of independent joint control (flexion synergy) in individuals with chronic moderate/severe upper-extremity impairment. Preclinical investigations have identified abduction loading during reaching exercise as a key therapeutic factor to improve reaching function. An augmentative approach may be to additionally target weakness by incorporating resistance training to increase constitutive joint torques of reaching with the goal of improving reaching function by “overpowering” flexion synergy. The objective was, therefore, to determine the therapeutic effects of horizontal-plane viscous resistance in combination with progressive abduction loading therapy.

**Methods:**

32 individuals with chronic hemiparetic stroke were randomly allocated to two groups. The two groups had equivalent baseline characteristics on all demographic and outcome metrics including age (59 ± 11 years), time poststroke (10.1 ± 7.6 years), and motor impairment (Fugl-Meyer, 26.7 ± 6.5 out of 66). Both groups received therapy three times/week for 8 weeks while the experimental group included additional horizontal-plane viscous resistance. Quantitative standardized progression of the intervention was achieved using a robotic device. The primary outcomes of reaching distance and velocity under maximum abduction loading and secondary outcomes of isometric strength and a clinical battery were measured at pre-, post-, and 3 months following therapy.

**Results:**

There was no difference between groups on any outcome measure. However, for combined groups, there was a significant increase in reaching distance (13.2%, effect size; *d* = 0.56) and velocity (13.6%, effect size; *d* = 0.27) at posttesting that persisted for 3 months and also a significant increase in abduction, elbow extension, and external rotation strength at posttesting that did not persist 3 months. Similarly, the clinical battery demonstrated a significant improvement in participant-reported measures of “physical problems” and “overall recovery” across all participants.

**Conclusion:**

The strengthening approach of incorporating horizontal-plane viscous resistance did not enhance the reaching function improvements observed in both groups. Data do not support the postulation that one can be trained to “overpower” the flexion synergy with resistance training targeting constitutive joint torques of reaching. Instead, flexion synergy must be targeted with progressive abduction loading to improve reaching function.

**Trial Registration:**

ClinicalTrials.gov, NCT01548781.

## Introduction

Robot-assisted therapies for upper-extremity stroke recovery are designed around the concept of high-dosage functional task practice (1). In fact, robotic approaches have been found in general to be effective at delivering higher dosage (2). However, systematic reviews provide equivocal evidence for improvements in both motor performance and functional capacity (2, 3). Perhaps a limiting factor of robot-assisted therapies is that they have largely ignored the specific underlying motor impairments constraining the functional tasks emulated by the robotic devices. The simplest functional tasks, such as reaching, are constrained by motor impairments that can be readily quantified. For example, in a simple functional task of reaching outward [to grasp an object], reaching distance and velocity are primarily limited by abnormal activation of elbow flexors that scale with shoulder effort (generation of abduction torque) (4). The abnormal co-activation of biceps brachii with deltoid (5) was originally observed and is still described clinically as “flexion synergy” (6, 7). It results in the loss of independent joint control (8, 9) or joint individuation (10) that is associated with both activity limitations and participation restrictions (11). Importantly, abnormal biceps activation occurs prior to the onset of elbow extension (flexor lengthening) distinguishing it from flexor spasticity, defined as hyperexcitable stretch reflexes (12), and overshadowing flexor spasticity as the primary contributor to reaching dysfunction in chronic stroke (4). Unfortunately, in the context of conventional robot-assisted therapies, massed practice of functional reaching with the limb fully supported by the device fails to address the expression of flexion synergy potentially impeding benefits to both motor performance and functional capacity explaining the equivocal therapeutic effects.

A paradigm shift in intervention approach is needed to further exploit the extensive capabilities of robot-assisted therapies. One solution would be to provide an environment for task practice and high dosage that optimizes the required neural drive to proximal shoulder abductors during performance of the reaching exercise. Preclinical investigations have attempted this and demonstrated success in reducing the impact of loss of independent joint control in both single-group (13) and randomized controlled dosage-matched studies (14) in individuals with moderate to severe chronic hemiparetic stroke. In these earlier studies, the robotic intervention, labeled as “progressive abduction loading therapy,” targeted the loss of independent joint control by progressively increasing the amount of required abduction effort during reaching practice across therapy sessions as performance (range of motion) improved. For example, at the onset of the intervention, a participant may have practiced reaching with the limb partially unweighted. As range of motion improved in subsequent sessions, the “abduction loading” was increased by making the limb heavier, even beyond that of normal gravitational loading in many participants. Importantly, the magnitude of abduction loading utilized in the intervention was standardized and participant-specific in that the shoulder abduction torque generation required during the reach was a percentage of their maximum isometric shoulder abduction strength. The studies found that in contrast to individuals who practiced reaching with abduction loading, individuals who practiced reaching without abduction loading (sliding along a horizontal table) did not improve in reaching distance under normal gravitational loading conditions. It was concluded that abduction loading was the key therapeutic factor in reaching practice and postulated that the amelioration of independent joint control occurred due to optimized utilization of residual neural resources such as ipsilesional corticospinal tract (14). Despite meaningful improvements, this approach did not fully restore reaching function warranting continued investigation of potential augmentative parameters.

In the present study, the therapeutic approach of progressive abduction loading therapy is expanded to investigate potential augmentative effects of incorporating resistance training. Weakness accompanies flexion synergy as one of the primary clinical signs in stroke-related hemiparesis (15). Therefore, targeting weakness while controlling for the expression of flexion synergy was postulated to have a complementary effect resulting in even greater gains in reaching function. The aim was, therefore, to determine if previously reported reaching improvements due to progressive abduction loading therapy (14) may be enhanced by specifically increasing the constitutive joint torques of horizontal planar reaching (elbow extension and horizontal adduction). Previous work has demonstrated that the incorporation of strength/power training, in the form of single-joint isokinetic exercise, with functional task practice improves isometric strength and dynamic joint power while enhancing improvements in activity limitations (16). This same approach has also demonstrated restorative over compensatory kinematic improvements such as improved elbow extension and reduced trunk displacement during a free reach to grasp task (17), providing evidence that an individual with stroke may be strengthened in such a fashion as to overpower flexion synergy and improve reaching range of motion. In the present study, the technical capabilities of the robotic device were exploited by integrating a strengthening element into the existing paradigm of progressive abduction loading therapy in the form of emulating a horizontal-plane viscous field. The viscous field safely resisted outward reaching motion. Participants reported subjectively that movement in the viscous field felt like attempting to reach through molasses. Prior methods of abduction loading therapy (14) were replicated such that reaching motion occurred at shoulder height with the whole arm in the horizontal plane. While horizontal planar reaching, and specifically horizontal adduction, does not precisely mimic natural free reaching, its utilization affords the ability to independently control for and manipulate parameters designed to target both weakness and flexion synergy during reaching movement. Namely, viscous resistance independently targeted elbow extension and shoulder horizontal adduction weaknesses while abduction loading independently targeted abnormal coupling of shoulder abduction and elbow flexion. It was hypothesized that the experimental incorporation of resistance training would act to strengthen elbow extension and horizontal adduction and, therefore, enhance the reaching range of motion improvements of progressive abduction loading therapy.

## Materials and Methods

### Study Design

A prospective, single-site, double-blinded (participant and evaluator), parallel comparison group, randomized clinical trial to determine the therapeutic effects of horizontal-plane viscous resistance in combination with progressive abduction loading therapy on reaching impairment, activity limitation, and participation restriction in individuals with chronic hemiparetic stroke was investigated and reported according to the CONSORT statement (18). All participants provided informed consent in accordance with the Declaration of Helsinki prior to participation in this study, which was approved by the Institutional Review Board of Northwestern University. This study is registered with ClinicalTrials.gov, identifier NCT01548781.

### Participants

**Figure 1:**
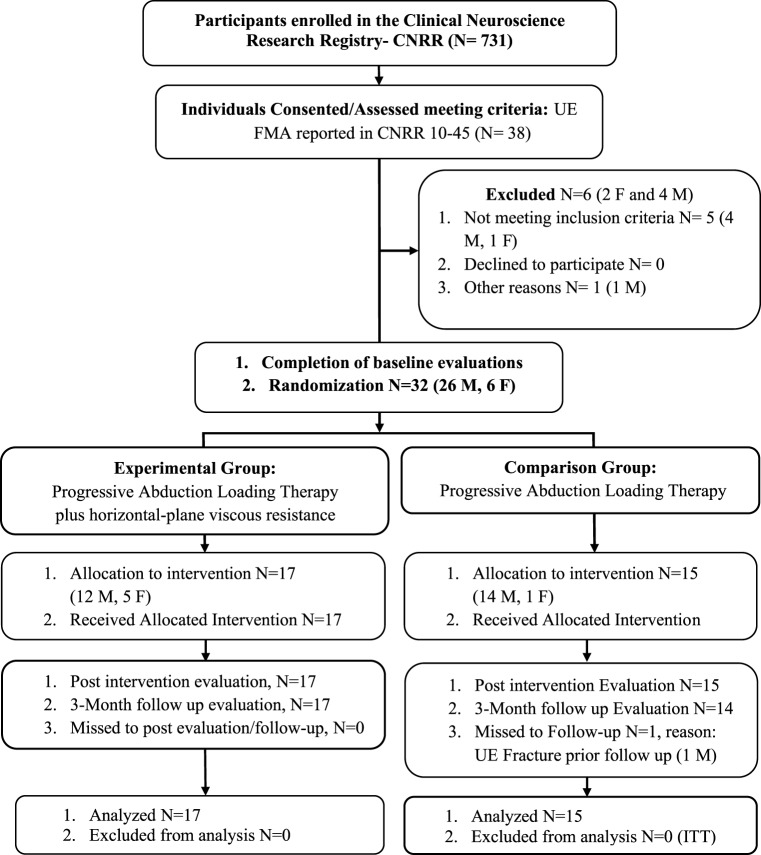
CONSORT flow diagram. Abbreviations: ITT, intention-to-treat; M, male; F, female; UE, upper extremity, FMA, Fugl-Meyer Assessment. Reasons for not meeting inclusion criteria: failed cognitive screen (2), low FMA (1), unsafe on robot due to motor impairment (2). Other reasons: participant moving permanently out of town (1).

All participants were recruited from a departmental research database under search criteria for score (10–45 out of 66) on the Upper Extremity Fugl-Meyer Motor Assessment (FMA) (19) (see Figure 1 for CONSORT flow diagram). Thirty-two individuals: 6 females and 26 males, ranging in age from 23 to 69 (mean: 59, SD: 11) and FMA baseline scores ranging from 16 to 43 (mean: 26.7, SD: 6.5) participated in the study (see Table 1 for all baseline comparisons). All participants had a self-reported clinical diagnosis of stroke-related hemiparesis (15 left- and 17 right-side) and were enrolled at least 1-year following stroke (mean: 10.1 years, SD: 7.6). Both groups were similar at baseline across all demographic and clinical data (Table 1). Inclusion criteria for the study were a FMA score within the range of 10–45 out of a possible 66. All participants were recruited and screened for inclusion in the study by a physical therapist blinded to the allocation of interventions. Potential participants were excluded if they had difficulty with sitting for long durations (self-report), recent changes in the medical management of hypertension (self-report), any acute or chronic painful condition in the upper extremities or spine, greater than minimal sensory loss in the affected arm as determined by a tactile localization and awareness of movement task (20), any motor impairment in the unimpaired limb (determined with FMA), and inability to follow a three-step command (21). Passive range of motion of the affected arm was measured to verify full passive extension of the elbow and at least 90° of passive shoulder motion in both the sagittal and coronal planes in order to participate in the study. Overpressure at the end of the range of motion was used as a medical screening procedure to verify the absence of an inflammatory condition at the upper-extremity joints.

**Table 1:** Mean (median, SD) baseline characteristics.

Characteristics	Total participants*N* = 32	Experimental group *N* = 17	Comparison group*N* = 15	Baseline comparison*P* value
Age in years	59 (62, 11)	59.8 (64, 15.6)	56.2 (58,12.9)	0.19^b^
Years since onset	10.13 (9, 7.61)	10.9 (9, 6.5)	11.1 (9, 6.1)	0.9^b^
Sex, *N* (%)				
Female	6 (18.8)	11 (64.7)	14 (93.3)	
Male	26 (81.2)	6 (35.3)	1 (6.7)	
Side of hemiparesis, *N* (%)				
Right	17 (53)	7 (41.2)	8 (53.3)	
Left	15 (46.9)	10 (58.8)	7 (46.7)	
Ethnicity, *N* (%)				
Hispanic	3 (9.38)	1 (5.9)	2 (13.3)	
Non-Hispanic	29 (90.6)	16 (94.1)	13 (86.7)	
Race, *N* (%)				
White	15 (46.9)	6 (35.3)	9 (60)	
Black	13 (40.6)	8 (47.1)	5 (33.3)	
American-Indian	0 (0)	0 (0)	0 (0)	
Asian	3 (9.4)	2 (11.8)	1 (6.7)	
Pacific Islander	0 (0)	0 (0)	0 (0)	
Non-reported	1 (3.1)	1 (5.9)	0 (0)	
Strength_shoulder horizontal adduction_	38.14 (37.71, 10.49)	35.15 (36.47, 7.65)	41.54 (37.85, 12.38)	0.15^b^
Strength_shoulder horizontal abduction_	22.62 (23.16, 7.93)	20.65 (20.71, 7.02)	24.84 (24.53, 8.53)	0.14^a^
Strength_shoulder abduction_	25.19 (24.40, 9.50)	24.11 (21.31, 9.35)	26.42 (25.11, 9.85)	0.50^a^
Strength_shoulder adduction_	33.11 (29.69, 12.87)	30.45 (27.20, 12.33)	36.12 (37.11, 13.21)	0.16^b^
Strength_shoulder external rotation_	8.17 (7.83, 4.89)	7.59 (6.44, 5.43)	8.82 (9.17, 4.28)	0.48^a^
Strength_ishoulder internal rotation_	11.67 (10.87, 5.58)	12.12 (12.77, 4.71)	11.16 (9.62, 6.56)	0.64^a^
Strength_elbow flexion_	26.78 (24.71, 12.57)	26.23 (20.71, 15.17)	27.40 (25.72, 9.27)	0.26^b^
Strength_elbow extension_	18.76 (17.77, 6.88)	17.87 (15.82, 6.27)	19.76 (18.79, 7.60)	0.45^a^
Reaching distance	0.69 (0.69, 0.28)	0.7 (0.78, 0.25)	0.67 (0.59, 0.32)	0.75^a^
Reaching velocity	0.81 (0.70, 0.46)	0.74 (0.71, 0.42)	0.88 (0.71, 0.52)	0.43^b^
UE FMA	26.70 (27, 6.5)	27.00 (27.00, 7.50)	26.33 (27, 5.29)	0.98^b^
FTHUE	4.44 (4, 0.88)	4.47 (4.00, 1.07)	4.40 (4.00, 0.63)	0.88^b^
MAL-28_AOU_	1.01 (0.62, 0.94)	1.02 (0.58, 1.16)	1.00 (0.89, 0.68)	0.44^b^
MAL-28_QOM_	1.00 (0.82, 0.73)	0.94 (0.8, 0.82)	1.06 (0.93, 0.65)	0.65^a^
SIS_Physical Problems_	0.45 (0.44, 0.15)	0.48 (0.50, 0.16)	0.43 (0.44, 0.13)	0.30^b^
SIS_Activities_	0.74 (0.76, 0.12)	0.72 (0.73, 0.14)	0.76 (0.78, 0.08)	0.26^a^
SIS_Mobility_	0.85 (0.86, 0.13)	0.84 (0.86, 0.11)	0.85 (0.89, 0.14)	0.49^b^
SIS_Hand_	0.21 (0.18, 0.17)	0.21 (0.20, 0.18)	0.20 (0.15, 0.16)	1.00^b^
SIS_Participation_	0.71 (0.70, 0.17)	0.71 (0.69, 0.19)	0.72 (0.72 0.14)	0.86^a^
SIS_Recovery_	61.8 (65.00, 18.00)	64 (70.00, 20.7)	59 (60, 14.42)	0.4^a^

*UE FMA, upper extremity Fugl-Meyer Assessment (max score 66); FTHUE, Rancho Los Amigos Functional Test for the Hemiparetic Upper Extremity; Strength Units, Newton-meters; Velocity and Distance, normalized data; MAL-28_AOU_, motor Activity Log-28–Amount of Use Scale; MAL-28_QOM_, motor Activity Log-28–Quality of Movement Scale; SIS, Stroke Impact Scale*.

*Baseline Comparisons*.

*^a^Values are derived from t-test*.

*^b^Values are derived from Mann–Whitney U test*.

### Study Setting, Randomization/Allocation, and Structure

The study took place at Northwestern University, Chicago beginning with recruitment on April 2012 and ending with the last follow-up on April 2015. Upon consenting and then passing screening, 32 participants were individually randomized, using a computer-generated program (MATLAB—Mathworks), and allocated to either the experimental or the comparison group (1:0.88 allocation ratio). The program was constructed prior to enrollment to balance the allocation of participants and their level of severity as measured by the FMA at baseline. The lower and upper cut-points for inclusion were 10 and 45, respectively (actual study range was 16–43 as reported above); therefore, the program contained five blocks that were equally stratified by potential FMA scores (10–16, 17–23, 24–30, 31–37, and 38–45). Each block contained four experimental and four comparison slots available for randomization. If at any time during enrollment a block would become full, it would be automatically increased by four experimental and four comparison slots. The randomization process was carried out by a study engineer who did not take part in any clinical component of the study (evaluation or intervention), and both the participants and the physical therapist evaluator were blinded to allocation. The participant’s FMA score was input into the algorithm by the study engineer and they were randomly allocated to group. Details of the allocation were sent electronically using an encrypted system to two treating physical therapists that carried out the intervention. Participants in the study were assessed three times by the evaluation physical therapist: pre- (baseline), post-intervention (8 weeks after beginning of training/end of the intervention), and 3 months following the end of the intervention. The treating physical therapists were blinded to all evaluation and clinical assessment results. For both groups, the intervention schedule consisted of 1-hour sessions, 3 times per week, for 8 weeks (total 24 sessions). Only on two occasions was the length of the intervention extended to 10 weeks to accommodate vacation/Christmas break. At least one intervention per week was provided during this period, allowing all participants to complete the intervention. There were no other variations to the protocol and no unanticipated problems or adverse events.

### Outcome Measures

The primary outcome measures were maximum reaching distance and peak endpoint reaching velocity. Kinematic evaluations were chosen as the primary outcomes since they offer a quantitative measurement of reaching function that account for the deleterious effects of flexion synergy (11, 22). To quantify changes in strength, the secondary outcome measure of isometric single-joint strength was utilized. Other secondary outcomes included, Fugl-Meyer Motor Assessment (19), Stroke Impact Scale (23), Motor Activity Log: amount of use (AOU) and quality of movement scales (QOM) (24), and Rancho Los Amigos Functional Test for the Hemiparetic Upper extremity (25). Kinematic/kinetic movement analyses are emphasized in order to provide greater insight into the mechanisms underlying the response to therapy. The objective of progressive abduction loading therapy is to restore normal movement in individuals with severe stroke as opposed to facilitating compensation. The outcome of reaching distance and velocity under standardized abduction loading provides a detailed evaluation of motor performance capable of quantifying restoration with high resolution (22).

#### Reaching Distance and Velocity

##### Positioning

**Figure 2:**
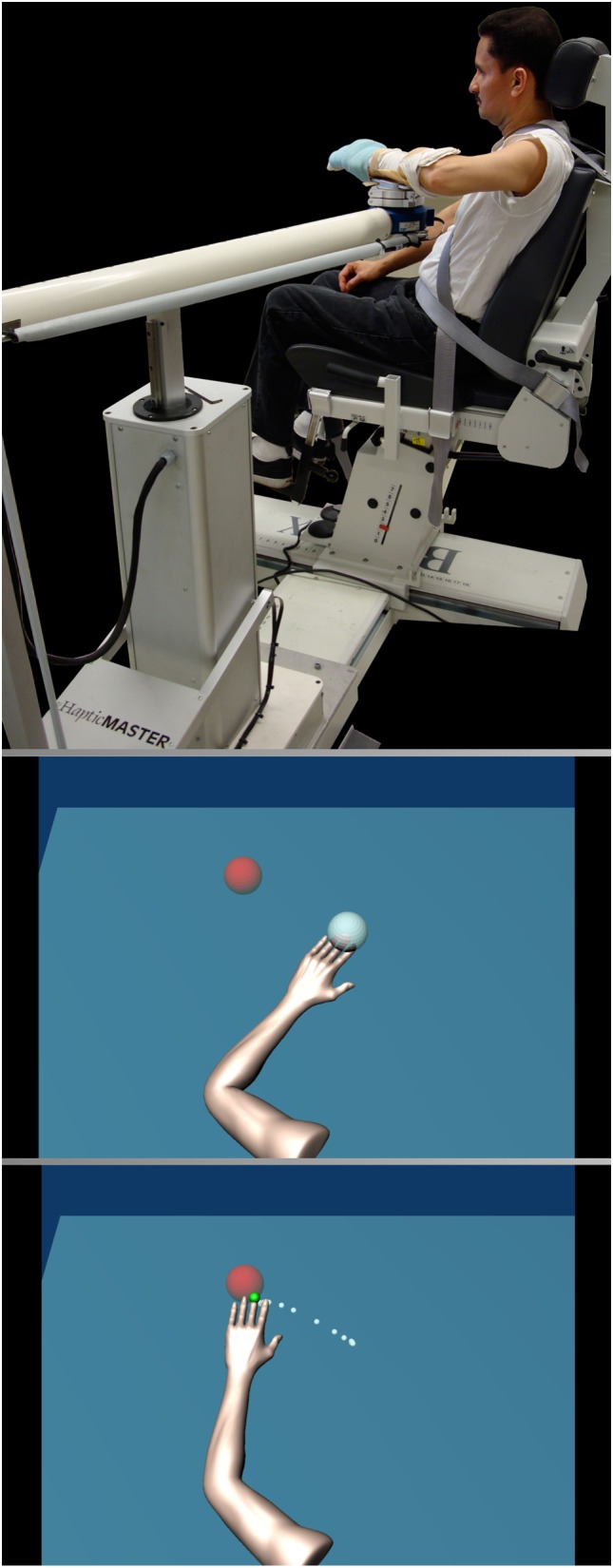
Participant set up (top). Written informed consent was obtained from the pictured participant for use in publication and education materials. Visual display (bottom) illustrating the arm avatar viewed by the participant including the home target (gray) and the reaching target (red) with the reaching trajectory shown in white dots.

Participants sat in a Biodex chair with their arm resting in a forearm-hand orthosis attached to the ACT^3D^, a mechatronic device used to provide precise abduction loading during reaching practice and evaluation (Figure 2, top) (22). The orthosis maintained the wrist and hand in a neutral position and the participant’s trunk was immobilized to prevent shoulder girdle movement by a set of straps attached to the experimental chair. The shoulder was positioned with the arm perpendicular or 90° to the line of gravity when the arm was resting on a haptically rendered table (virtual table maintained by the device and displayed using visual feedback). Additionally, the participant’s arm was positioned in 40° of horizontal adduction and 110° of elbow flexion. This position is referred to as the “home position” in subsequent narrative. The standardized home position, in combination with measured limb segment lengths, is utilized by the ACT^3D^ software to calibrate a graphic representation of the arm and illustrate it on a computer screen in front of the participant (Figure 2, bottom).

##### Reaching without Abduction Loading

Once positioned and supported by the haptic table, participants were asked to view the feedback monitor and slide their hand along the haptic table acquiring and maintaining in the home position (gray circle in Figure 2). After the endpoint of the hand acquires the home position, data collection begins. 1 s after data collection is initiated, a second circle representing the movement target appears on the screen as a cue for the participant to begin the movement (red circle in Figure 2). The movement target is located requiring an additional 100° of elbow extension and 30° of horizontal adduction from the home position to acquire. Participants were instructed to move as rapidly as possible toward the target and to maintain the final position until disappearance of the target (end of data collection). Rapid (ballistic) movements were strongly encouraged through verbal cuing by the experimenter. The avatar of the participant’s arm emulated movement in real-time providing realistic visual feedback of movement performance. During the completion of each target reach, the hand path was displayed to the participant as feedback. The length of data collection was 5 s per trial and ten consecutive trials were performed.

##### Reaching with Abduction Loading

**Figure 3:**
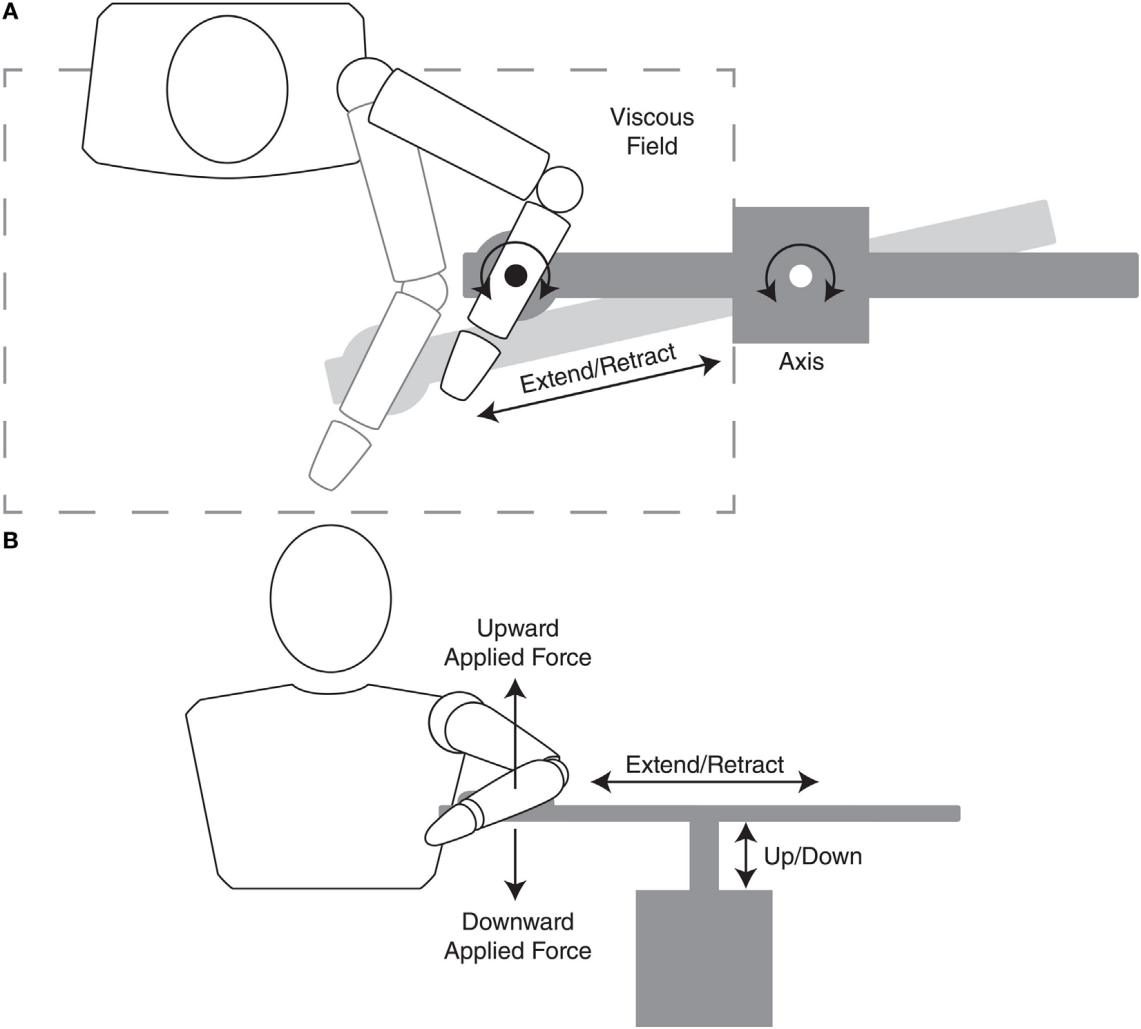
Diagram of top **(A)** and front **(B)** views illustrating the interface of the participant and device. The top view illustrates both the kinematics (extend/retract and axis rotation motion) and viscous resistance of the device in the horizontal plane during an outward reaching motion involving elbow extension and shoulder horizontal adduction. The front view illustrates both kinematics (extend/retract and up/down motion) and kinetics (upward and downward force) of the device. Regarding the frontal-plane kinetics, during a reaching task, if the required volitional abduction torque (abduction loading) was greater than the weight of the limb, the device would emulate a downward force making the limb heavier. In contrast, if the required abduction torque was less than the weight of the limb, the device would emulate an upward force partially unweighting the limb.

Participants were then asked to repeat the ballistic reaching movements while maintaining specific levels of shoulder abduction loading. Figure 3 illustrates how the ACT^3D^ provided vertical force while the participant performed the reaching task in the horizontal plane. During reaching with abduction loading trials, participants were required to lift the arm off of the haptic table prior to acquiring the home position. The haptic table was lowered slightly such that, when lifting off of the haptic table, the arm achieved 90° of abduction. This abduction *joint position* was maintained throughout the reaching movement while the ACT^3D^ provided a constant vertical force. Due to the mechanics of the equipment, the volitional abduction torque required to lift the arm off of the haptic table was also maintained constant. Importantly, the vertical force provided by the ACT^3D^ was calculated so that the volitional abduction torque was equivalent to a standardized percentage of their shoulder abduction strength. These percentages represented the different abduction loading conditions that were tested and included 0, 12.5, 25, 37.5, and 50% of max abduction strength. It is important to note that the volitional abduction torque required for a given abduction loading condition may be more than the abduction torque required to lift the arm against gravity. In that circumstance, the vertical force provided by the ACT^3D^ would be directed downwards as illustrated in Figure 3. In the opposing circumstance where the volitional torque required was less than the torque required to lift the limb against gravity, the vertical force provided by the ACT^3D^ would be directed upwards. For all participant assessments, one set of 10 repetitions were performed for each of the five randomized abduction loading conditions. Therefore, at completion of the primary outcome assessment, participants performed six sets of 10 reaches (one without abduction loading and five with abduction loading). In order to prevent fatigue there was ~5–15 s of rest between repetitions and ~1–3 min of rest between each set.

#### Isometric Strength

Isometric strength was measured via maximum voluntary torque (MVT) for eight joint torque directions, including shoulder abduction/adduction, horizontal adduction/abduction, internal/external rotation, and elbow flexion/extension using methods described in detail in our previous work (13, 14, 26, 27). Participants were positioned in exact fashion as in the assessment of reaching performance with their arm/hand in the home position. The forearm and hand were immobilized in fiberglass casting material and rigidly fixed to a six degree-of-freedom load cell [see Figure 1 of Ellis et al. (28) for an image of the experimental setup/device]. Maximum voluntary joint torque was measured in random order for each joint torque direction by asking the participant to “pull/push as hard as you can” using real-time visual feedback of the specific torque direction. MVT was defined as the single largest joint torque obtained within a maximum of six trials such that three trials were within 10% of magnitude and the last trial was not the largest. Maximum voluntary abduction torque was used for the standardized abduction loading levels as part of the evaluation of reaching performance described above and intervention protocol described below.

### Intervention Protocol

Subjects sat in the experimental chair, with their paretic arm in the “home position” on a horizontal haptic surface or “table”(Figure 2, top). The experimental and comparison interventions were similar in that they consisted of reaching movements to four standardized target locations while lifting an optimized percentage of the maximum voluntary shoulder abduction torque replicating the initial preclinical studies (13, 14). The reaching target locations were intentionally biased toward elbow extension requiring 100° of extension from the home position compared to 0°, 20°, 40°, and 60° of horizontal adduction from the home position for targets 1–4 respectively. The focus on elbow extension was designed to facilitate strengthening of elbow extension. For both the comparison and experimental groups, the initial level of shoulder abduction loading for each target direction was optimized as the highest abduction load at which the participant could reach at least 50% of the distance to the target. Participants were trained at this abduction loading level until they could reach at least 80% of the distance to the target in 8 out of 10 repetitions for three out of four sets. The abduction load was then increased to the highest level where, again, participants could reach at least 50% of the distance between the starting position and the target. The levels of abduction load were increased at intervals of 12.5% of their individual pretesting abduction MVT.

For the experimental group, a viscous field was added in the horizontal plane after the abduction loading was determined for all four targets. The viscous field provided a velocity-dependent increase in resistance for movement in the horizontal plane. Vertical movement was not impacted by the viscous field. Subjects were then trained using the same progression as the comparison group, as stated above. In both groups, shoulder abduction loading was progressed independently for each target. Verbal feedback of movement performance was provided to the participants in both groups by the physical therapist during the sessions to help participants familiarize with the exercise and to let them know how far they reached. For both groups, each intervention session consisted of four sets of 10 repetitions for each of the four target directions totaling 160 repetitions. Rest periods of up to 10 s between repetitions and a fixed 1-min rest between sets were provided to avoid fatigue and overuse injury. The order of the 16 sets was randomized for each session. The time of each session was truncated at 1 h to emulate outpatient rehabilitation.

### Sample Size

The sample size was obtained by calculating the effect size, *d*, from our initial pilot study that collected similar kinematic data (14). Effect size (*d*) was calculated by dividing the difference between group means (*X*_1_ − *X*_2_) by the common SD (*s*) following confirmation of homogeneity of variance between the two groups. Therefore, for *d* = (*X*_1_ − *X*_2_)/*s, d* = ((0.09 m^2^) − (−0.01 m^2^))/0.09 = 1.18. The power for our previous work for detecting a significant (*a* = 0.05) difference between groups with two groups of seven participants (*n* = 14) was 0.93. For the present study, if we assume an effect size of 1.18 represented a clinically meaningful change in reaching kinematics, a total of 20 participants (10 per group) would be necessary to detect a significant (*a* = 0.05) improvement in response to the proposed intervention with a power of 0.98. We completed two groups (15, 17) totaling 32 participants in an effort to increase the generalizability of the study findings to the severe stroke population and address a potential attrition rate of 25% based on our experience in the initial pilot study (14).

### Data Analysis

**Figure 4:**
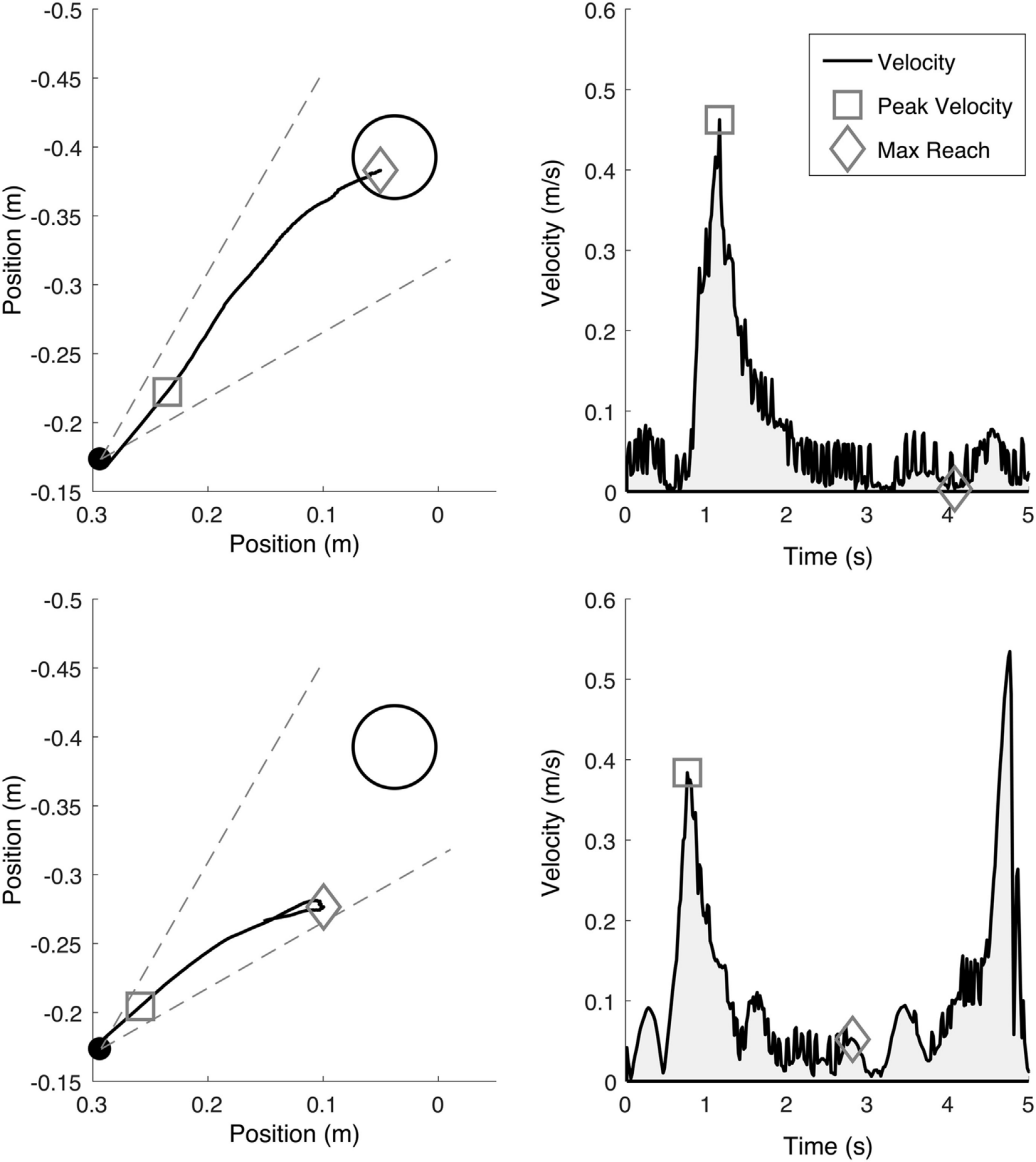
Example maximum reaching trajectories (left, indicated by a diamond) while supported on a haptic horizontal surface (top) and while abducting off of the horizontal surface at 50% of maximum abduction strength (bottom). Reaching trajectories were coached to be as fast and as far as possible in the direction of the target and only accepted if within the ±15° cone of tolerance (gray dotted line). The endpoint peak reaching velocity (right, indicated by a square) illustrates the peak reaching velocity associated with each reaching trajectory.

Position data from the ACT^3D^ were recorded at a sampling rate of 50 Hz and saved offline for future analysis of the primary outcomes of maximum reaching distance (22) and peak reaching velocity. A custom data processing program was implemented in MATLAB to determine maximum reaching distance and velocity for each reaching trial [see “Methods” in Ellis et al. (4), for a detailed description]. Reaching distance was calculated as the most distant position of the reaching trajectory that remained within the tolerance of a 30° cone centered in the direction of the reach target and while the arm remained off of a horizontal haptic surface positioned at shoulder height (Figure 4). The largest reaching distance was identified from the five reaching trials of the highest abduction loading level that the participant was able to complete during the pretesting session. Maximum reaching distance was normalized to the absolute distance from the home position to the reaching target, each standardized to shoulder/elbow joint configuration. The same analysis was completed at the same abduction loading level for subsequent post- and 3-month follow-up testing sessions.

Reaching velocity was calculated from endpoint position (middle finger tip) as the first peak tangential reaching velocity for each reaching trial. The first peak was utilized as it represented the maximum effort to initiate outward movement against synergy from a standardized position and controlled abduction load (Figure 4). Subsequent velocity peaks, usually slower yet still contributing to outward reaching distance, were not utilized as they are confounded by factors such as muscular endurance and the effects of possible greater end-range passive muscle stiffness following stroke (29). The largest reaching velocity was identified from the same five reaching trials utilized for calculating reaching distance. Reaching velocity was normalized by maximum reaching velocity achieved during a reach while supported on the haptic surface.

Isometric strength was calculated using the joint torque data with a custom data processing program in Matlab. Torque data was first filtered with a 0-phase 250-ms moving average window. Next, the instantaneous peak torque was identified within each trial. The largest overall peak torque of all trials for each tested torque direction was defined as the maximum isometric strength.

Digital data for the secondary outcomes were exported from FileMaker Pro 12.0v5 (FileMaker, Inc.) to Microsoft Excel for Mac Version 15.34 in preparation for statistical analysis.

### Statistical Methods

All dependent variables were analyzed with IBM SPSS Statistics Version 24 and Matlab R2016b with an alpha-level of 0.05. An intention-to-treat approach was implemented using a last-observation-carried-forward strategy for 1 participant that did not complete the 3-month follow-up assessment. Baseline data were first evaluated for both normality and homoscedasticity using Lilliefor’s Test and Two-sample *F*-test, respectively. Baseline between-group comparisons used Mann–Whitney *U* Test or 2-tailed Student’s *t*-test with or without equal variance depending on results for normality and homoscedasticity. For the primary outcomes, normality of distribution was determined using quantile–quantile plots. Sphericity was assessed using Maukley’s Test. In cases where sphericity was not assumed, the Greenhouse–Geisser correction was utilized and indicated with the subscript, “G-G,” when reporting *F*-statistics. An analysis of variance on a two-way mixed-design model with repeated measures was utilized to test the effect of session (pre-, post-, and follow-up) and group (comparison and experimental) and the interaction effect of session × group for both primary outcomes of reaching distance and velocity and the secondary outcome of isometric strength (*N* = 8 torque directions). *Post hoc* comparisons were completed when significant effects were found. Second, between-group comparisons on change scores were also completed for primary outcomes of reaching distance and velocity using Mann–Whitney *U* test or two-tailed Student’s *t*-test. In cases where homoscedasticity was not assumed, the degrees of freedom were adjusted and indicated with the subscript, “a,” when reporting *t*-statistics.

For secondary outcomes, change scores were analyzed for both normality and homoscedasticity. Between-group comparisons of change scores were performed for both pre- to posttesting and pre- to 3-month follow-up testing using Mann–Whitney *U* test or two-tailed Student’s *t*-test. Specific to only the Fugl-Meyer Assessment, the number of participants per group with change scores of 5 or greater were tabulated in a similar fashion to a recent and related large-scale clinical trial (30) to illustrate the proportion of responders/non-responders in each group. The change score of +5 represents the reported value for both minimal detectable change (31) and clinically important difference (32). Additionally, combined-group analysis of total Fugl-Meyer Assessment scores was completed using a Friedman test of differences among repeated measures (session) followed by *post hoc* comparison with Wilcoxon Paired-Sample Sign Rank Test.

## Results

### Baseline Data

There were no significant differences between groups at baseline for any demographic or outcome variable (see Table 1).

### Reaching Distance

**Figure 5:**
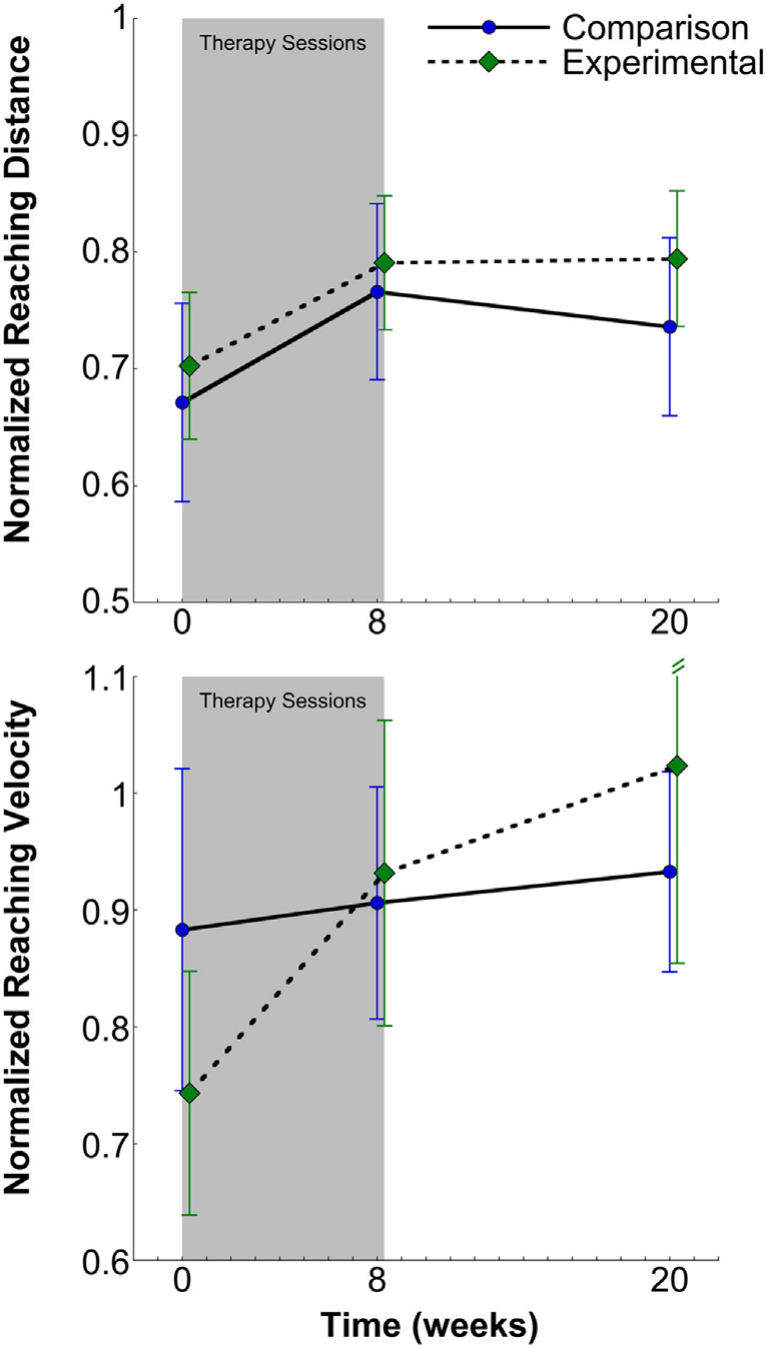
Normalized reaching distance (top) and velocity (bottom) and SEs for the comparison and experimental groups at pretesting, posttesting, and 3-month follow-up. There was a significant increase in reaching distance and velocity for all participants at posttesting that persisted at 3-month follow-up in all participants.

For the primary outcome of normalized reaching distance under maximum abduction loading conditions, the two-way analysis of variance yielded a main effect for session, *F*(2,60) = 5.97, *p* = 0.004 (Figure 5, top). Both the main effect of group, *F*(1,30) = 0.19, *p* = 0.669 and the interaction effect of session × group, *F*(2,60) = 0.19, *p* = 0.826 were non-significant. *Post hoc* testing for the main effect of session indicated that, for all participants, mean (±SE) reaching distance increased 13.2% at posttesting [0.78 ± 0.05, *t*(31) = 3.18, *p* = 0.003, 95% CI [0.69, 0.87], effect size; *d* = 0.56] compared to pretesting (0.69 ± 0.05, 95% CI [0.59, 0.79]) and persisted at 3-month follow-up [0.77 ± 0.05, *t*(31) = 2.59, *p* = 0.015, 95% CI [0.67, 0.86], effect size; *d* = 0.46]. Change scores for reaching distance were subjected to parametric analysis and indicated no significant difference between the experimental and comparison groups for pre- to posttesting [experimental; 0.09 ± 0.03, comparison; 0.10 ± 0.06, *t*(21.4_a_) = 0.11, *p* = 0.92, 95% CI [−0.12, 0.13]] and for pre- to 3-month follow-up testing [experimental; 0.09 ± 0.03, comparison; 0.06 ± 0.06, *t*(30) = 0.43, *p* = 0.67, 95% CI [−0.15, 0.10]].

### Reaching Velocity

The primary outcome of normalized peak reaching velocity was not normally distributed, so non-parametric analyses were utilized. Mann–Whitney *U* Test indicated no difference between groups across sessions (experimental; 0.86 ± 0.07, 95% CI [0.71, 1.01], comparison; 0.87 ± 0.06, 95% CI [0.75, 0.99], *z* = −0.56, *p* = 0.58). Friedman Test indicated a significant effect of session [χ^2^(2) = 16.46, *p* < 0.001] for combined groups (*N* = 32) (Figure 5, bottom). Mean (±SE) reaching velocity for both posttest (0.92 ± 0.08, *z* = −2.39, *p* = 0.02, 95% CI [0.76, 1.08], effect size; *d* = 0.27) and 3-month follow-up (0.98 ± 0.10, *z* = −3.25, *p* = 0.001, 95% CI [0.79, 1.17], effect size; *d* = 0.32) were 13.6 and 21% greater, respectively, than pretesting (0.81 ± 0.08, 95% CI [0.64, 0.98]). Change scores for peak reaching velocity were subjected to non-parametric analysis and indicated no significant difference between the experimental and comparison groups for pre- to posttesting (experimental; 0.19 ± 0.08, 95% CI [−0.24, 0.29], comparison; 0.02 ± 0.13, 95% CI [0.02, 0.36], *z* = −0.38, *p* = 0.71) and for pre- to 3-month follow-up testing (experimental; 0.28 ± 0.15, 95% CI [−0.02, 0.58], comparison; 0.05 ± 0.13, 95% CI [−0.21, 0.31], *z* = 0.00, *p* = 1.00).

### Isometric Strength

The secondary outcome of isometric strength was subjected to a two-way analysis of variance having three levels of session (pre, post, 3-month follow-up) and two levels of group (comparison, experimental) for each of the eight torque directions. See Table 2 for compiled summary results. The two-way analysis of variance yielded a main effect of session for combined groups (*N* = 32) for shoulder abduction, *F*(1.62_G-G_, 47.04_G-G_) = 9.11, *p* = 0.001 elbow extension, *F*(1.39_G-G_, 40.33_G-G_) = 4.28, *p* = 0.033 and external rotation, *F*(2,58) = 3.94, *p* = 0.025 such that isometric strength increased 14, 12, and 19% respectively from pre- to posttesting. Significant differences did not remain at 3-month follow-up testing. The two-way analysis of variance yielded a main effect of session for combined groups for shoulder extension, *F*(1.57_G-G_, 45.41_G-G_) = 5.62, *p* = 0.011, however, demonstrating a decrease of 11% from post- to 3-month follow-up testing. The only main effect of group was for horizontal adduction, *F*(1,29) = 4.23, *p* = 0.049 indicating that horizontal adduction was 17% greater on average across all sessions in the comparison group. However, there were no main effects for session or interaction effect of session × group for horizontal adduction indicating the comparison group was generally stronger in this degree-of-freedom but not enough to show a baseline difference. Finally, the only interaction effect of session × group was for elbow flexion, *F*(2,2) = 4.21, *p* = 0.02 indicating that elbow flexion in the comparison group increased 13% from pre- to posttesting.

**Table 2:** Mean (SD) isometric single-joint strength.

Joint torque	Pre		Post		3-month follow-up	
	COM	EXP	COM	EXP	COM	EXP
Elbow flexion	27.4 (9.3)	26.2 (15.2)	31.0 (8.2)^c^	24.4 (11.4)	30.7 (8.1)	24.6 (12.2)
Elbow extension	19.8 (7.6)	17.9 (6.3)	23.7 (7.7)^a^	18.8 (7.3)^a^	23.2 (7.2)	18.1 (7.0)
Abduction	26.4 (9.9)	24.1 (9.3)	30.2 (7.4)^a^	27.6 (11.2)^a^	29.0 (9.1)	23.3 (10.1)
Adduction	36.1 (13.2)	30.4 (12.3)	39.5 (13.4)	29.6 (12.7)	36.9 (12.1)	29.9 (11.1)
Horizontal adduction	41.5 (12.4)^b^	35.1 (7.7)	44.4 (11.2)^b^	36.4 (10.8)	41.8 (14.1)^b^	35.2 (9.0)
Horizontal abduction	24.8 (8.5)	20.7 (7.0)	24.6 (9.8)	22.1 (7.2)	21.2 (9.9)^a^	19.4 (7.6)^a^
External rotation	8.8 (4.3)	7.6 (5.4)	10.3 (4.9)^a^	9.2 (6.3)^a^	9.7 (3.5)	8.6 (5.9)
Internal rotation	11.2 (6.6)	12.1 (4.7)	11.5 (5.8)	13.6 (6.1)	11.7 (6.2)	13.8 (7.4)

*COM, comparison group; EXP, experimental group*.

*^a^Significant between-session difference (pre vs post OR pre vs follow, combined groups)*.

*^b^Significant between-group difference (CON vs EXP, combined sessions)*.

*^c^Significant within-group difference (pre vs post)*.

### Clinical Battery

Pre- to posttest and pre- to 3-month follow-up change scores were not significantly different between groups for the secondary outcome measures (clinical battery). These data are compiled in Table 3. For the secondary analysis of the Fugl-Meyer Motor Assessment, there was a significant effect of session on total score [χ^2^(2) = 17.15, *p* < 0.001] when combining groups (*N* = 32). Furthermore, Wilcoxon Sign-Ranks test indicated that both mean (±SE) posttest scores (28.9 ± 1.2, *z* = −3.52, *p* = 0.001) and 3-month follow-up scores (29.5 ± 1.3, *z* = −3.75, *p* = 0.001) were significantly greater than pretest scores (26.7 ± 1.2). Individual change-score tabulation of responders indicated that three participants in each group (experimental, 17%; comparison, 20%) achieved a change score of ≥5 points at posttest, and six participants in each group (experimental, 35%; comparison, 40%) achieved a change score of ≥5 points from pretest to 3-month follow-up.

**Table 3:** Mean (SD) secondary outcome change scores.

Outcome measures	Pre to post change		Pre to 3-month follow-up change	
	COM	EXP	COM	EXP
UE FMA	2.13 (3.07)	2.29 (2.85)	2.60 (3.64)	2.94 (3.09)
FTHUE	0.00 (0.53)	0.12 (0.49)	−0.07 (0.59)	0.12 (0.78)
MAL-28_AOU_	−0.16 (0.32)	−0.04 (0.47)	−0.27 (1.15)	−0.02 (0.38)
MAL-28_QOM_	−0.09 (0.36)	0.07 (0.48)	−0.14 (0.74)	−0.03 (0.39)
SIS_Physical Problems_	0.06 (0.11)	0.08 (0.12)	0.13 (0.21)	0.07 (0.16)
SIS_Activities_	0.02 (0.08)	0.02 (0.08)	−0.00 (0.13)	0.02 (0.07)
SIS_Mobility_	0.02 (0.13)	0.03 (0.10)	0.02 (0.10)	0.02 (0.09)
SIS_Hand_	0.05 (0.17)	0.04 (0.09)	0.05 (0.16)	0.02 (0.11)
SIS_Participation_	0.02 (0.14)	0.05 (0.14)	0.06 (0.17)	0.03 (0.14)
SIS_Recovery_	0.07 (0.08)	0.03 (0.12)	0.04 (0.14)	0.04 (0.12)

*COM, comparison group; EXP, experimental group; FTHUE, Rancho Los Amigos Functional Test for the Hemiparetic Upper Extremity; MAL-28AOU: motor Activity Log-28–Amount of Use Scale*.

## Discussion

The main finding of this RCT is negative in that the hypothesis of enhanced reaching function and isometric strength with the incorporation of resistance training to standard progressive abduction loading therapy was not supported. The addition of horizontal-plane viscous resistance did not enhance the gains in reaching function or strength that were experienced by the comparison group receiving standard progressive abduction loading therapy. The rationale of incorporating resistance training was to specifically target and increase constitutive reaching torques of elbow extension and shoulder horizontal adduction thus conferring a therapeutic benefit of enhanced gains in reaching function. Instead, both groups improved in isometric strength and reaching function, but without a between-groups effect. The gains in single-joint strength experienced by both groups are consistent with previous studies (16, 17) in that significant improvements were realized in the trained directions. In the present intervention, the trained directions were the individual joint motions required to complete the reaching exercise including abduction, shoulder horizontal adduction, and elbow extension. However, in the present study there was no additional benefit of incorporating resistance training. This is inconsistent with previous work where single-joint power training augmented functional task practice (17). Perhaps in the present study, the multi-joint nature of the resistance was not sufficient to have the same effects as single-joint power training. An additional difference in the present study was that isometric strength gains were not retained at 3-month follow-up testing as were the gains in reaching function. The dissociation of strength and reaching function gains diminishes the likelihood of changes in single-joint isometric strength contributing to changes in reaching function. This is important in the context of rehabilitating reaching dysfunction in that it suggests targeting the impairment of flexion synergy over that of weakness.

With the improvements in reaching function experienced by both groups persisting 3 months following therapy despite the ceasing gains in isometric strength, it suggests that improvements in reaching function may have been due to targeting flexion synergy as opposed to weakness of constitutive reaching torques. These results confirm previous work (14) that systematic progression of abduction loading is a key therapeutic factor in ameliorating reaching function. In the present study, it was demonstrated that while isometric strengthening is possible in the trained directions, it is not necessary in order to realize improvements in reaching function. Instead, the primary impairment limiting reaching function poststroke may be the loss of independent joint control (flexion synergy) that emerges when activating shoulder abductor muscles (4, 8, 9, 33, 34). Reaching function was evaluated in a manner that accounted for the expression of flexion synergy impairment but did not quantify flexion synergy *per se*. Reaching range of motion is known to diminish monotonically as a function of increasing abduction load—this function defining the expression of flexion synergy (9). By measuring reaching distance and velocity at a single functionally relevant abduction load (the heaviest abduction load the participant could complete at baseline), the primary outcome captures reaching function while accounting for the expression of flexion synergy. The persistence of improvements in reaching function vs strength may also be explained by the additional mobility during activities of daily living afforded by improved independent joint control. Increased mobility may translate to increased functional use in activities whose parameters are capable of perpetuating gains in independent joint control but not sufficient to maintain gains in strength. That said, and considering the persistence of reaching function but not isometric strength, these data suggest that impairments of independent joint control and strength appear to traverse separate recovery trajectories that require different behavioral/environmental stimuli to be maintained.

Implications of the lack of enhancing effects of horizontal-plane viscous resistance are particularly relevant to rehabilitation device development. Future devices or equipment developed to implement progressive abduction loading therapy need not be overly sophisticated with the only requirement being that they are capable of delivering progressive abduction loading spanning the range of fully unweighting the arm to more than doubling the weight of the arm (22). Participants in this study trained at abduction loads spanning 25–100% of pretesting maximum abduction strength representing near full gravitational unweighting to increasing limb weight several fold. This capability is lacking in current leading therapeutic equipment on the market such as the *Saebo*Mas (Saebo Inc., North Carolina) and Armeo^®^ Spring (Hocoma, Switzerland) that are limited to only unweighting (gravitational compensation). Moreso, the unweighting force of these devices is not uniform throughout the horizontal plane. The addition of controlled progressive abduction loading capabilities beyond limb weight may enhance the positive therapeutic effects of task practice with gravity compensation found with these devices (30) allowing outcomes to more convincingly surpass conventional care.

This study included standardized clinical outcomes to reflect conventional methods in stroke recovery research, however, were positioned as “secondary outcomes.” This positioning reflected the objective of the study that was to evaluate restoration of movement with high resolution avoiding criticisms of conventional interventions such as constraint-induced movement therapy that have been argued to facilitate task-specific compensation as opposed to restoring normal movement (35). Significant improvement in the Fugl-Meyer Motor Assessment was realized but without a between-groups effect reflecting the primary outcomes. It is impossible to distil from the Fugl-Meyer Assessment score whether improvements in loss of independent joint control, single-joint strength, or both contributed to gains. Here, the sole sustained improvements were in reaching function that were measured kinematically under standardized abduction loading. This provides compelling quantitative data indicating that loss of independent joint control may be ameliorated and may better explain improvements in reaching function than changes in strength.

Patient reported outcomes were included to measure the perceptions of study participants. In the present study, participants’ perception, as measured by absolute score on the Stroke Impact Scale, found the domains of “physical problems” and “overall recovery” improved from baseline in both groups (Table 3). This indicates that both groups perceived improvement regardless of grouping. Conversely though, self-report of the actual AOU of the arm, as measured by the Motor Activity Log, did not change from baseline. An explanation of this discrepancy may be that with the perception of improved function, changes in the AOU are less well noticed. Observation of actual AOU as opposed to self-report may provide clarification but was not investigated in this study. Future work may also investigate the implementation of strategies such as a transfer package (36) to increase actual AOU during daily activities and capitalize on gains in reaching function to ameliorate activity limitations.

A primary limitation to this study was the inability to blind the intervention therapists from the intervention. While they were blinded to all other processes, they could not be blinded to the administration of horizontal-plane viscous resistance in the experimental group. Biases from this knowledge were reduced through the employment of standardized procedure. The viscous field was standardized as well as parameters for progression of the intervention as discussed in Section “Materials and Methods” above. Additionally, all participants were given real-time visual feedback of performance and regular verbal coaching and encouragement to enhance performance during therapy sessions. While a device, *per se*, may not be necessary to administer progressive abduction loading therapy, its use affords quantitative control not only reducing bias but also increasing efficiency. A second limitation to the study may be the sample size and associated confidence intervals. However, independent of sample size, the pooled effect size of *d* = 0.56 is telling of a meaningful improvement of all participants in reaching function. It should be acknowledged though that while the effect size is “medium” or even “large enough to be visible to the naked eye” (37), it is not as large as the original pilot study [*d* = 1.18 (14)]. The original pilot study included a control group of reaching exercise without abduction loading illustrating the large beneficial effect of abduction loading whereas in the present study both groups received abduction loading. It is very meaningful then that even in a pooled pre-post comparison of two groups receiving abduction loading therapy that a medium effect size was realized. Additionally, the sample size is more than double that of the pilot study increasing its generalizability.

Evidence for improved and persistent reaching function and patient-perceived improvement in recovery in individuals with chronic stroke not only advocates for the translation of this knowledge to chronic stroke rehabilitation but also to the future investigation of efficacy in early stroke recovery (in-patient rehabilitation). The rationale for application of progressive abduction loading therapy in early recovery stems from our understanding of the neuroanatomical underpinnings of loss of independent joint control. In brief, the leading hypothesis for the impairment of loss of independent joint control is that following a substantial loss of ipsilesional corticospinal tract, there is an increased reliance on contralesional corticoreticulospinal tract (28, 38–40). As a consequence, the multi-segmental branching of this motor tract results in concurrent activation of large groups of muscles, such as shoulder abductors and elbow, wrist, and finger flexors (5, 41, 42), producing a loss of independent joint control (8). Activation of this “backup” motor system is thought to be dynamic and driven by task demands requiring progressive increases in proximal joint muscle activation (4, 22). Considering the underlying neural circuitry, improvements in reaching performance observed in the present study point most logically to improved utilization of residual ipsilesional corticospinal tract that affords independent joint control.

The question then arises; why are residual corticospinal tracts not being utilized at full potential in chronic stroke? The answer to this may be conceived in early recovery during the critical period (43) of neuroplasticity. Brain edema resulting from the death of neural substrates following a stroke may initially reduce access to residual corticospinal tracts. As a result, intervention approaches may inadvertently facilitate the activation of contralesional corticoreticulospinal tract through many common therapeutic activities that intensely drive the motor system such as gait, stairs, and transfer training. Torque generation during even simple tasks in the lower extremity have been shown to produce involuntary flexion posturing of the affected arm (44). Measurements of reaching function in early recovery (in-patient rehabilitation) show the immediate expression of flexion synergy in all patients but with a varying time course of recovery with some achieving no recovery at all (45). Therefore, novel interventions will need to offset any potential negative effects by directly targeting the utilization of residual ipsilesional corticospinal tract to control the affected arm. Results of this study suggest that progressive abduction loading therapy may be a viable option for not only optimizing utilization of ipsilesional corticospinal tract but also facilitating persistent effects. Future work should investigate the short and long term effects of progressive abduction loading therapy in in-patient rehabilitation including current imaging approaches (40) capable of confirming this postulation.

## Ethics Statement

This study was carried out in accordance with the recommendations of Northwestern University Institutional Review Board with written informed consent from all subjects. All subjects gave written informed consent in accordance with the Declaration of Helsinki. The protocol was approved by the Northwestern University Institutional Review Board.

## Author Contributions

ME acquired outcome data, analyzed and interpreted data, and lead the drafting of the manuscript. JD and CC conducted the therapeutic interventions and edited the manuscript. JPAD interpreted the data and edited the manuscript. All authors read and approved the final manuscript.

## Conflict of Interest Statement

The authors declare that the research was conducted in the absence of any commercial or financial relationships that could be construed as a potential conflict of interest.
